# Supplementary material for: Chinese Herbal Extracts Mitigate Ammonia Generation in the Cecum of Laying Hens: An In Vitro Study
Source: Animals (Basel). 2023 Sep 20;13(18):2969. doi: 10.3390/ani13182969 (PMC10525658; doi:10.3390/ani13182969)
Supplement: Supplementary file 1 [file animals-13-02969-s001.zip › animals-2530644-supplementary.pdf]

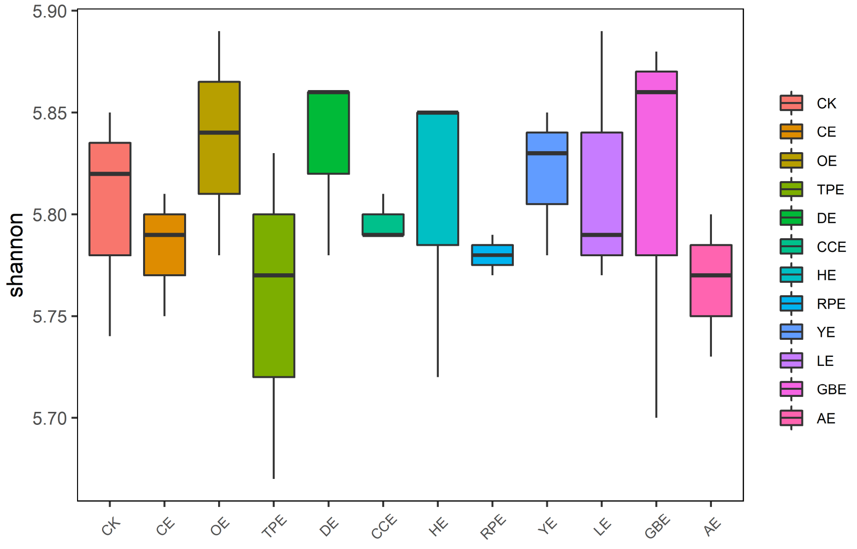

Figure S1. Effect of different types of Chinese herbal extracts on shannon index of fermentation bacteria. CK-control group, CE-Cinnamon Extract, OE-Osmanthus Extract, TPE-Tangerine Peel Extract, DE-Dandelion Extract, CCE-Coptis Chinensis Extract, HE-Honeysuckle Extract, PRE-Pulsatilla Root Extract, YE-Yucca Extract, LE-Licorice Extract, GBE-Ginkgo Biloba Extract, AE-Astragalus Extract.

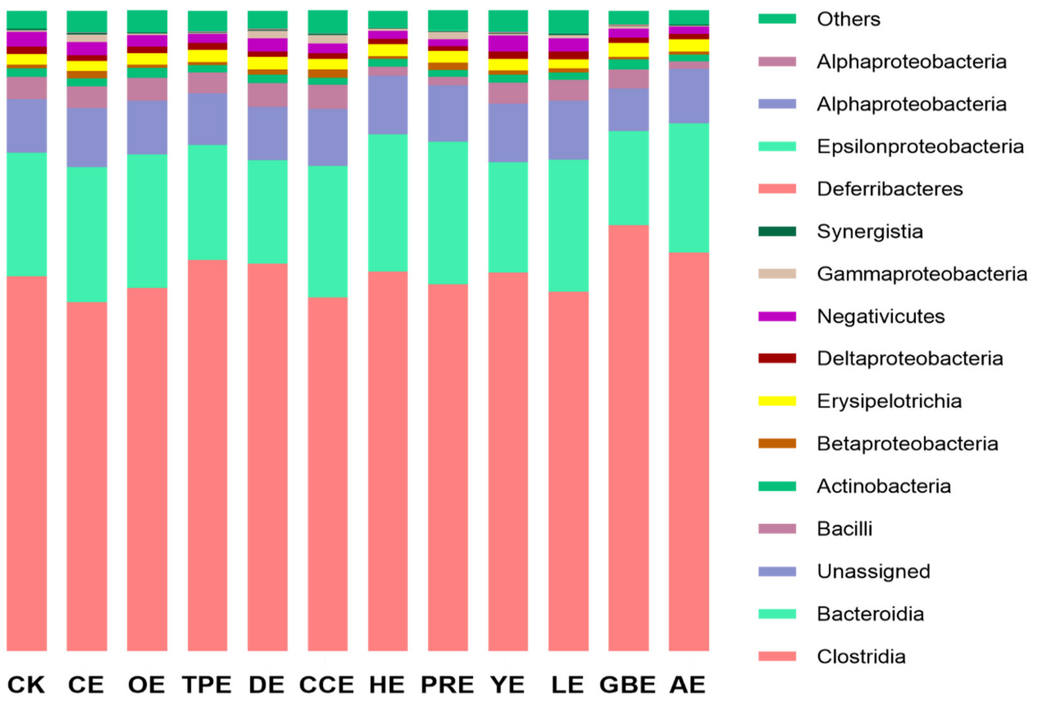

Figure S2. Effect of different types of Chinese herbal extracts on the class of the microbial community. CK-control group, CE-Cinnamon Extract, OE-Osmanthus Extract, TPE-Tangerine Peel Extract, DE-Dandelion Extract, CCE-Coptis Chinensis Extract, HE-Honeysuckle Extract, PRE-Pulsatilla Root Extract, YE-Yucca Extract, LE-Licorice Extract, GBE-Ginkgo Biloba Extract, AE-Astragalus Extract.

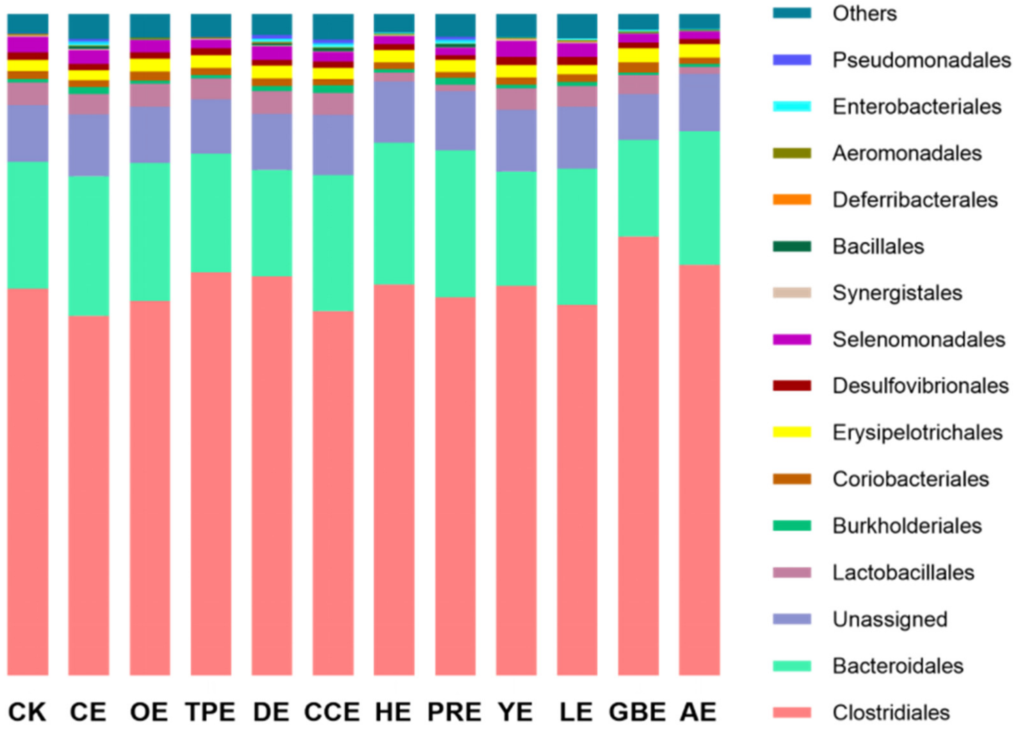

Figure S3. Effect of different types of Chinese herbal extracts on the order of the microbial community. CK-control group, CE-Cinnamon Extract, OE-Osmanthus Extract, TPE-Tangerine Peel Extract, DE-Dandelion Extract, CCE-Coptis Chinensis Extract, HE-Honeysuckle Extract, PRE-Pulsatilla Root Extract, YE-Yucca Extract, LE-Licorice Extract, GBE-Ginkgo Biloba Extract, AE-Astragalus Extract.

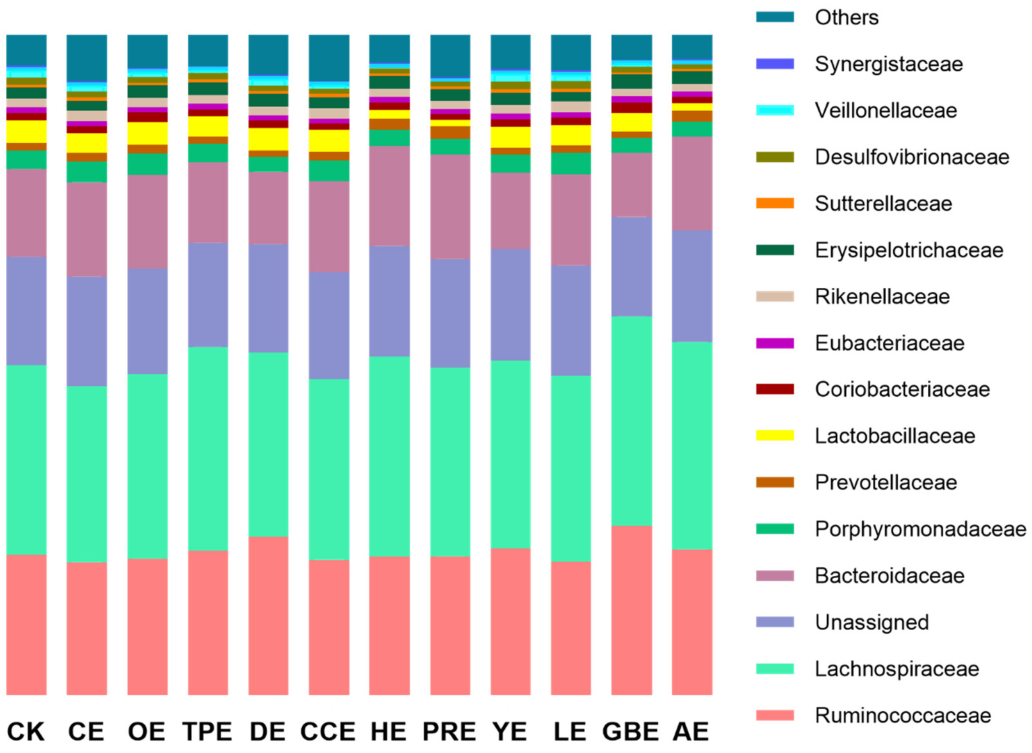

Figure S4. Effect of different types of Chinese herbal extracts on the family of the microbial community. CK-control group, CE-Cinnamon Extract, OE-Osmanthus Extract, TPE-Tangerine Peel Extract, DE-Dandelion Extract, CCE-Coptis Chinensis Extract, HE-Honeysuckle Extract, PRE-Pulsatilla Root Extract, YE-Yucca Extract, LE-Licorice Extract, GBE-Ginkgo Biloba Extract, AE-Astragalus Extract.
